# The efficacy and safety of the addition of poly ADP-ribose polymerase (PARP) inhibitors to therapy for ovarian cancer: a systematic review and meta-analysis

**DOI:** 10.1186/s12957-020-01931-7

**Published:** 2020-07-04

**Authors:** Yingzhu Yang, Nannan Du, Laidi Xie, Jing Jiang, Jiahang Mo, Jiaze Hong, Danyi Mao, Derry Minyao Ng, Huiwei Shi

**Affiliations:** 1 Department of Gynecology, Ningbo Women and Children’s Hospital, Liuting Street 339, Haishu District, Ningbo, Zhejiang, 315000 China; 2 The Second Clinical Medical College, Zhejiang Chinese Medical University, Hangzhou, Zhejiang China; 3 Zhejiang University, School of Medicine, Hangzhou, Zhejiang China; 4 Basic Medical College, Zhejiang Chinese Medical University, Hangzhou, Zhejiang China; 5 Medical College of Ningbo University, Ningbo, Zhejiang China

**Keywords:** PARP inhibitor, Ovarian cancer, BRCA 1/2, HRD, Meta-analysis

## Abstract

**Background:**

The purpose of this study was to explore the efficacy and tolerability of poly ADP-ribose polymerase (PARP) inhibitors in patients with ovarian cancer.

**Methods:**

The meta-analysis searched the PubMed, Web of Science, EBSCO, and Cochrane libraries from inception to February 2020 to identify relevant studies. And the main results of this study were long-term prognosis and treatment-related adverse events.

**Results:**

The results showed that the addition of PARP inhibitors could significantly prolong progression-free survival (PFS) and overall survival (OS) for patients with ovarian cancer (HR 0.44, 95% CI 0.34–0.53, *p* < 0.001; HR, 0.79, 95% CI 0.65–0.94, p < 0.001, respectively). In the BRCA 1/2 mutation patients, the HR of PFS was 0.29 (*p* < 0.001), and the HR was 0.51 (p < 0.001) in the no BRCA 1/2 mutation patients. The HR of PFS was 0.40 (*p* < 0.001) in the homologous recombination deficiency (HRD) mutation patients, while the HR was 0.80 (*p* < 0.001) in the no HRD mutation patients. Moreover, the analysis found that the use of PARP inhibitors did not significantly increase the risk of all grade adverse events (AEs) (RR = 1.04, p = 0.16). But the incidence of grade 3 or higher AEs was increased (RR = 1.87, p = 0.002). In general, the AEs were mainly manifested in the blood system.

**Conclusions:**

PARP inhibitors can improve the prognosis of ovarian cancer patients with and without genetic mutations (BRCA 1/2 or HRD). Furthermore, PARP inhibitors were tolerable to patients when added to their current therapy, although it inevitably adds the grade 3 and higher AEs.

## Background

Ovarian cancer was the fourth most common cause of cancer-related deaths in women, with an estimated 200,000 cases and 125,000 deaths worldwide each year [1]. More than two thirds of the patients were diagnosed as advanced. Over 90% of malignant ovarian tumors were of epithelial origin and were called epithelial ovarian cancer. The most common and deadly epithelial ovarian cancer was high-grade serous ovarian cancer [2]. Moreover, the prognosis for the disease was poor: the 5-year survival rate standardized for the European average age was only 37.6% between 2000 and 2007 [3]. For the past decade, standard treatments for women with advanced ovarian cancer have been surgery and platinum-based chemotherapy. Some researchers have tried to improve this standard two-drug chemotherapy by adding a third cytotoxic drug, and the results have shown that it failed to affect progression-free survival or overall survival and resulted in increased toxicity [4–6]. In patients with newly diagnosed advanced ovarian cancer, the standard option was to add bevacizumab, an antiangiogenic agent, to carboplatin plus paclitaxel, and then bevacizumab alone [7, 8]. However, the long-term efficacy of this medication was still uncertain.

On the other hand, studies have shown that PARP (poly ADP-ribose polymerase) was a key regulator of DNA damage repair, and PARP enzymes played a vital role in repairing single-strand breaks through base excision repair [9]. In double-strand break repair, PARP facilitated homologous repair and inhibited less conserved non-homologous and micro-homologous end-junction repair. Without PARP, homologous repair will not work and the less conservative repair process will dominate [10]. PARP inhibitors captured PARP on the DNA of the single-strand break site, thereby preventing the repair of these breaks and producing double-strand breaks, which were not accurate in tumors with homologous recombination deficiency (HRD) repair [11]. Recently, in some randomized clinical trials, the use of PARP inhibitors as a single drug or in combination with other drugs in patients with advanced ovarian cancer (including BRCA mutation or HRD mutation or neither mutation) can benefit this population to varying degrees [12–14]. Currently, some researchers have proposed four mechanisms for inhibiting PARP: (1) it inhibited PARP by inhibiting base excision repair. (2) It could capture PARP on damaged DNA [15], which interfered with the catalytic cycle of PARP, hindered DNA repair, and promoted double-strand breaks. (3) It could destroy the recruitment of BRCA1 to damaged DNA to achieve the effect of inhibiting PARP. (4) It could also activate non-homologous end connections to inhibit PARP [16].

In addition, studies have shown that PARP inhibitors can prevent cancer cells from repairing damaged DNA and leading to cancer cell death [17]. Its role was to capture the repair complex at the site of single-strand breaks, preventing replication and causing further damage [18]. In fact, the DNA damage induced by PARP inhibitors affected the process of mitosis. The detailed mechanism was that PARP inhibitors will damage the stability of the replication fork and cause DNA damage, which will be transferred to mitosis. During mitosis, these DNA damages could lead to chromatin bridges and lead to cytokinesis failure, multinucleation, and cell death. Other studies have shown that PARP inhibitors could promote the induction of cell death through the process of mitosis and eliminate the cytotoxicity caused by PARP inhibitors in some ways [19]. However, for patients with BRCA 1/2 unmutated and HRD unmutated, the efficacy of PARP inhibitors was still uncertain. The purpose of this study was to explore the efficacy and tolerability of PARP inhibitors in patients with ovarian cancer.

## Methods

### Search strategy

The meta-analysis searched the PubMed, Web of Science, EBSCO, and Cochrane libraries from the beginning to February 2020 to identify relevant studies. Because the authors have different calling habits for the same drug in the literatures, we choose a combination of free-text terms and medical subject heading terms which was used for topic search to ensure the integrity of the included literatures. Search terms included “PARP inhibitor,” “poly ADP-ribose polymerase inhibitor,” “olaparib,” “rucaparib,” “veliparib,” “niraparib,” “talazoparib,” “iniparib,” “ovarian neoplasms,” “ovarian cancer,” “ovarian carcinoma,” “carcinoma of ovary,” or “ovary cancer.” Also, we manually search for references in the literature. In order to make the study more standardized and its conclusions more scientific, this meta-analysis was conducted following the guidelines of the Preferred Reporting Items for Systematic Review and Meta-Analysis Protocols (PRISMA-P) 2015 statement [20].

### Inclusion and exclusion criteria

Inclusion criteria: (1) included articles were English reports of completed clinical controlled trials evaluating the efficacy of PARP inhibitors to ensure the completeness of included studies. (2) The included articles were randomized controlled trials (RCTs) for PARP inhibitors in phase II and phase III for patients with ovarian cancer in order to increase the credibility of the research conclusion. (3) Included articles mentioned PFS (progression-free survival) and/or OS (overall survival) so that we could effectively analyze the results.

Exclusion criteria: (1) research treatments that included neoadjuvant therapy. (2) The trial was not a phase II or phase III clinical trial. (3) Research data cannot be extracted. (4) In the event where the author has repeated publications or continuous updates, we would use the latest article.

### Outcome measures

The main results of this study were long-term prognosis (including PFS and OS) and treatment-related adverse events (AEs), because these two types of indicators played a vital role in the prognosis of patients.

The incidence of AEs was characterized based on all grades and grade 3 or higher as reported by each trial using the definitions of National Cancer Institute’s Common Terminology Criteria for Adverse Events (CTCAE) version 4. 0[21].

### Assessment of the risks of bias and data extraction

We assessed the potential risks of bias in trials by using the Cochrane Collaboration Risk of Bias Assessment tool [22]. In order to ensure the objectivity and accuracy of the entered data, two investigators completed the review independently. A third investigator resolved disagreements. The basic information of each study was independently extracted by two researchers. The baseline information included author, publication period, the number of ClinicalTrials.gov, the phase of clinical trials, the regimen of the experimental group of using a PARP inhibitor and control group, the number of participants, tumor mutation, first-line or non-first-line treatment, and survival benefit indicators including PFS, OS, and AEs. If different studies contain different chemotherapy regimens, we can try to conduct a subgroup analysis when the sample size is sufficient. However, if the chemotherapy regimens are variable and the samples are insufficient, we carefully combine the corresponding results.

### Statistical analysis

We compared the PFS and OS of the experimental group of using a PARP inhibitor and the control group, expressed as hazard ratio (HR) and 95% confidence interval (CI), and the AEs involved were also expressed as risk ratio (RR) and 95% CI. All pooled results are displayed using the forest plots. We used Begg’s and Egger’s test with a level of significance set at *P* < 0.1 to evaluate publication bias and the Stata 12.0 Software to perform the sensitivity bias [23]. Because this study included a variety of treatments, we used a random-effects model in order to increase credibility. P value less than 0.05 was considered statistically significant.

## Results

### Eligible studies and inclusion features

After rigorous searching and selection, including manual searching, we have identified 1247 potentially relevant articles. In the excluding neoadjuvant therapy, not a PARP inhibitor compared to other, non-phase II or III studies and data unavailable after, the study included ten related articles ultimately [12–14, 24–30]. The minute search and selection process are detailed in Supplementary Figure 1. These included studies involving three clinical phase II trials and seven clinical phase III trials, which covered a total of 5006 patients. Of which, PARP inhibitors were used as a non-first-line treatment in six studies and as first-line treatment in four studies. In the choice of treatment therapy, six studies chose the treatment regimen of PARP inhibitors versus placebo. Four experimental groups of studies treated with PARP inhibitors in combination with other drugs. In the selection of PARP inhibitors, most studies (6) chose olaparib. Among these objects of study, three studies incorporated the population of BRCA 1/2 mutation. At the end of the study results, all studies reported PFS and five studies reported OS. Detailed research characteristics are shown in Supplementary Table 1.

### Efficacy of PARP inhibitors on the PFS and OS

**Fig. 1:**
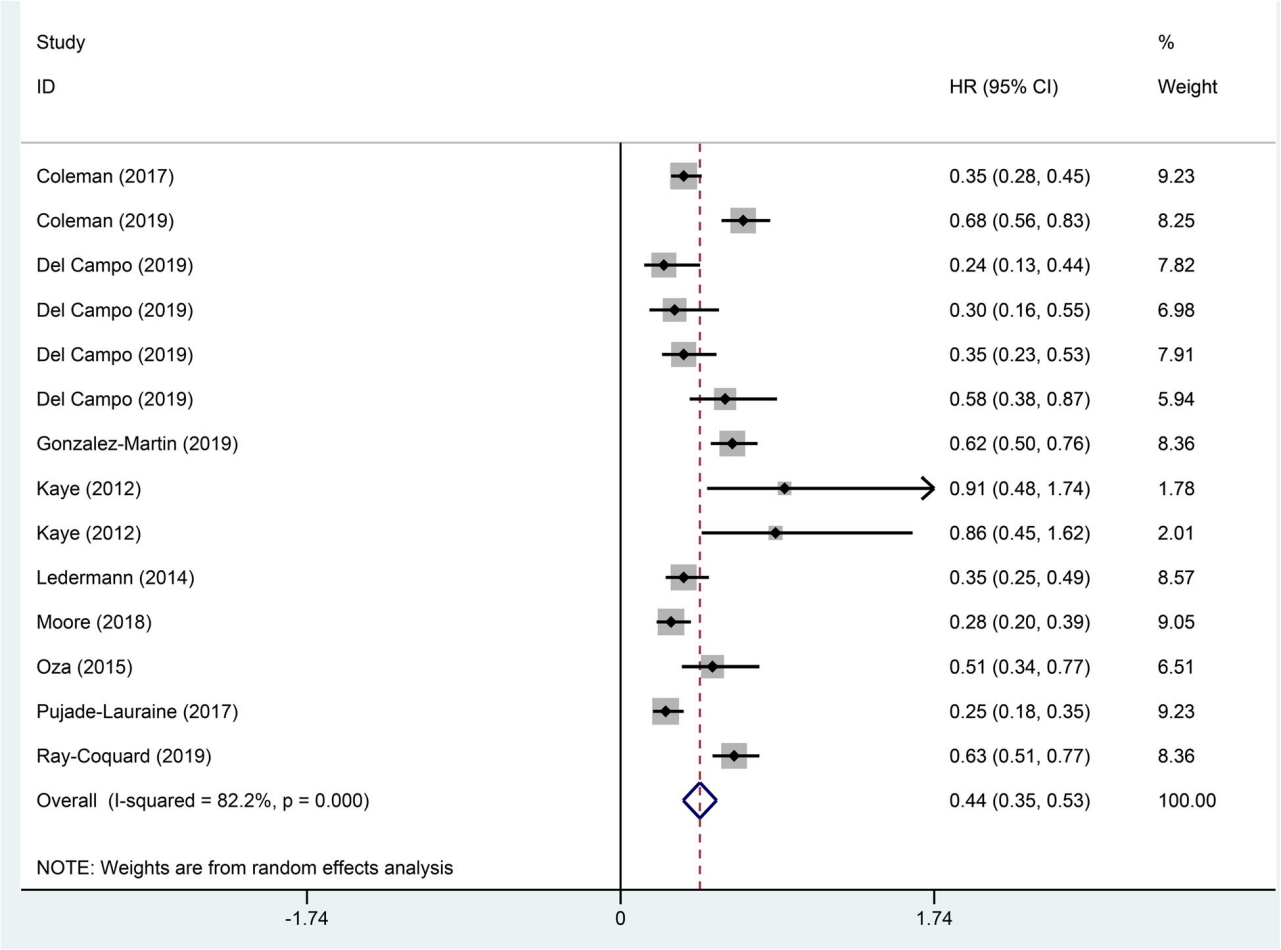
Forest plots of the progression-free survival (PFS) for additional PARP inhibitors for patients with ovarian cancer (p < 0.001)

**Fig. 2:**
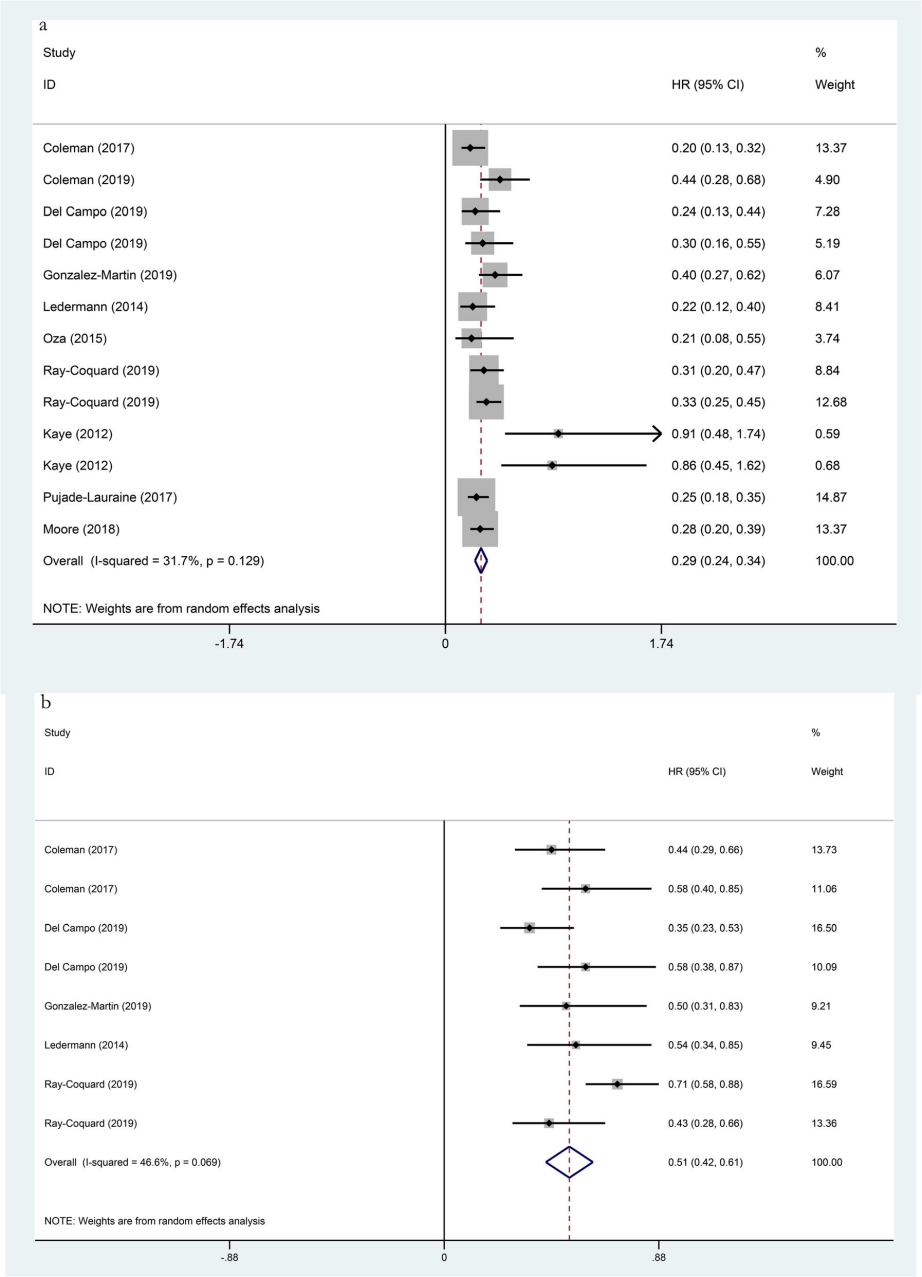
Forest plots of the progression-free survival (PFS) for additional PARP inhibitors. **a** BRCA 1/2 mutation (*p* < 0.001). **b** no BRCA 1/2 mutation (*p* < 0.001)

**Fig. 3:**
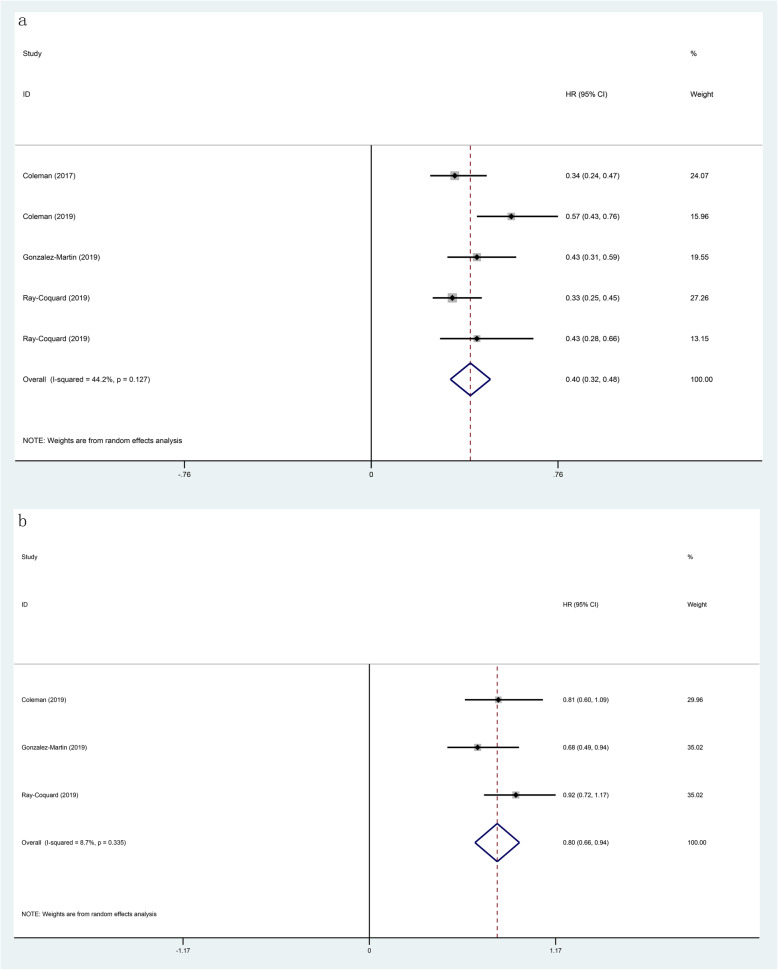
Forest plots of the progression-free survival (PFS) for additional PARP inhibitors. **a** HRD mutation (*p* < 0.001). **b** no HRD mutation (*p* < 0.001)

In this included study, all of them reported on PFS, which the results showed that the use of PARP inhibitors could significantly prolong PFS (HR 0.44, 95% CI 0.34–0.53, p < 0.001; Fig. 1). Besides, we performed a subgroup analysis of the included population, which had a genetic mutation or not. The analysis found the classification method of two gene mutations in the included population, in which one was the BRCA 1/2 mutation and the other was the HRD mutation. In the BRCA 1/2 mutation patients, the HR of PFS was 0.29 (95% CI 0.24–0.34, *p* < 0.001; Fig. 2), and the HR was 0.51 (95% CI 0.42–0.61, *p* < 0.001; Fig. 2) in the no BRCA 1/2 mutation patients. The HR of PFS was 0.40 (95% CI 0.32–0.48, *p* < 0.001; Fig. 3) in the HRD mutation patients, while the HR was 0.80 (95% CI 0.66–0.94, *p* < 0.001; Fig. 3) in the no HRD mutation patients. Studies have shown that the PFS of people with BRCA 1/2 mutation or HRD mutation was significantly longer than no gene mutation.

**Fig. 4:**
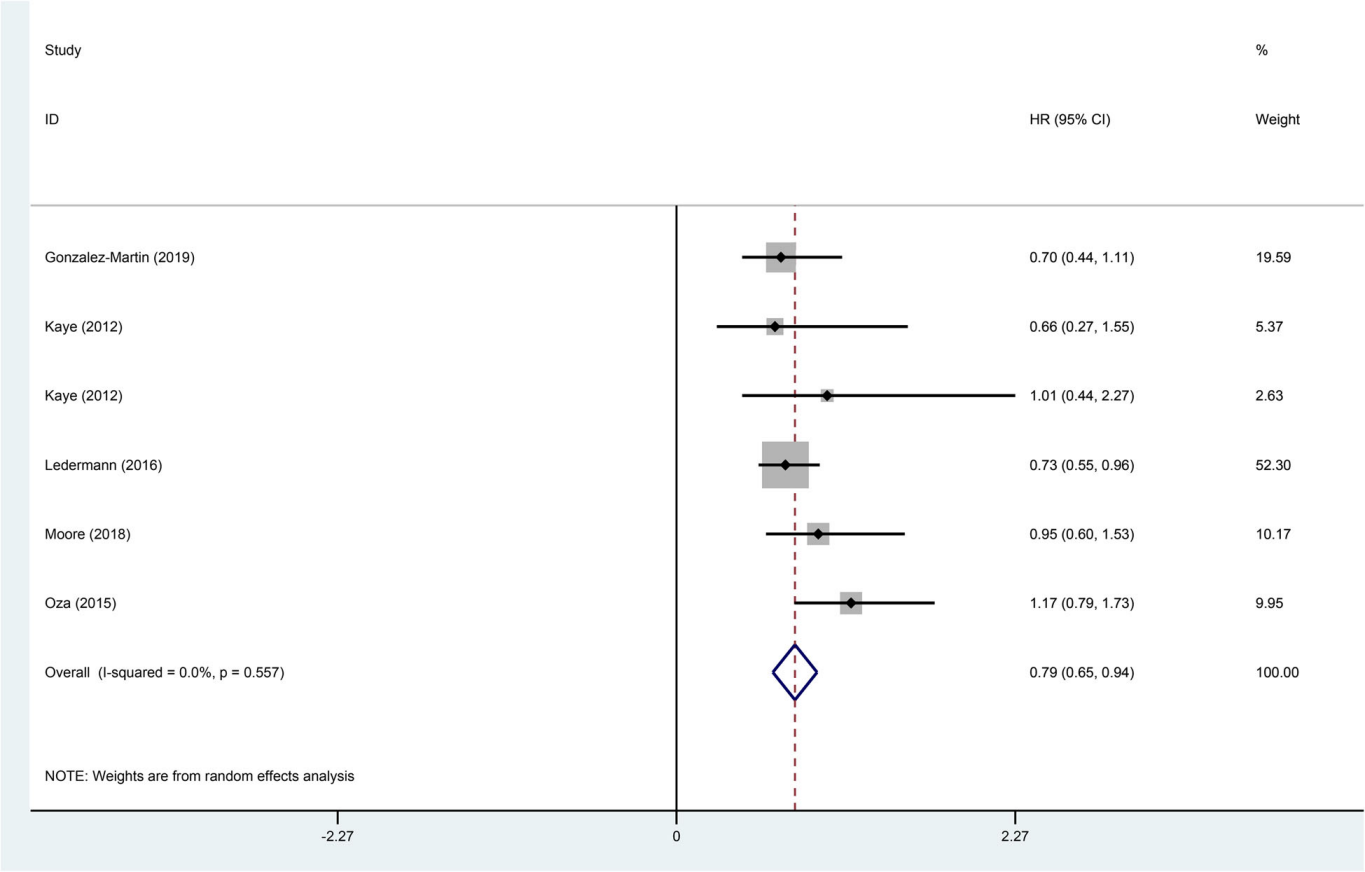
Forest plot of the overall survival (OS) for additional PARP inhibitors for patients with ovarian cancer (*p* < 0.001)

In this study cohort, there were a total of five studies that mentioned OS. Estimates of HR and at 95% CI were weighted and pooled using the random effect model. The results showed that the experimental groups using PARP inhibitors could significantly prolong OS compared with the control group (HR 0.79, 95% CI 0.65-0.94, *p* < 0.001; Fig. 4). Also, the heterogeneity was low.

### Analysis of the adverse events of PARP inhibitors

The analysis found that the use of PARP inhibitors did not significantly increase the overall risk of AEs (RR = 1.04, p = 0.16). Anemia and thrombocytopenia were the most common AEs, with risk values of 3.40 (95% CI 1.86–6.19, *p* < 0.001) for anemia and 3.59 (95% CI 1.65–7.78, *p* < 0.001) for thrombocytopenia. The other AEs were different such as decreased appetite (RR = 1.45), diarrhea (RR = 1.23), dizziness (RR = 1.46), fatigue (RR = 1.36), headache (RR = 1.35), nausea (RR = 1.71), neutropenia (RR = 1.88), and vomiting (RR = 1.82). Among grade 3 and higher AEs, the overall risk increased (RR = 1.87, *p* = 0.002). Among the various AEs, anemia and thrombocytopenia were the more significant increase, and their risk values ​were 9.94 (95% CI 2.63–37.50, *p* < 0.001) for anemia and 4.46 (95% CI 1.26–17.19, *p* = 0.02) for thrombocytopenia. The other AEs were fatigue (RR = 2.39), nausea (RR = 2.57), and neutropenia (RR = 2.42). In general, the AEs were mainly manifested in the blood system. Detailed data are shown in Tables 1 and 2.

**Table 1:** Subgroup analysis of the any grade adverse events

Experimental vs. control	No. of studies	RR	95% CI	p	Heterogeneity
					(I^2^) (%)
Any grade adverse events	8	1.04	0.99–1.10	0.16	97
Any grade abdominal pain	9	0.97	0.84–1.12	0.68	36
Any grade anemia	9	3.40	1.86–6.19	< 0.001	95
Any grade arthralgia	7	0.94	0.82–1.07	0.32	0
Any grade constipation	9	1.16	0.91–1.49	0.22	75
Any grade decreased appetite	6	1.45	1.21–1.74	< 0.001	7
Any grade diarrhea	8	1.23	1.07–1.40	0.003	20
Any grade dizziness	6	1.46	1.11–1.94	0.008	36
Any grade fatigue	9	1.36	1.20–1.53	< 0.001	65
Any grade headache	8	1.35	1.04–1.74	0.02	68
Any grade nausea	9	1.71	1.35–2.17	< 0.001	92
Any grade neutropenia	8	1.88	1.23–2.87	0.003	89
Any grade thrombocytopenia	7	3.59	1.65–7.78	0.001	91
Any grade vomiting	9	1.82	1.48–2.23	< 0.001	60

**Table 2:** Subgroup analysis of the grade 3 or higher adverse events

Experimental vs. control	No. of studies	RR	95% CI	p	Heterogeneity
					(I^2^) (%)
Grade 3 or higher adverse events	8	1.87	1.27–2.77	0.002	96
Grade 3 or higher abdominal pain	9	1.08	0.69–1.68	0.74	0
Grade 3 or higher anemia	10	9.94	2.63–37.50	< 0.001	92
Grade 3 or higher arthralgia	7	0.81	0.32–2.03	0.66	0
Grade 3 or higher constipation	9	0.92	0.31–2.72	0.88	16
Grade 3 or higher decreased appetite	6	2.39	0.76–7.52	0.14	0
Grade 3 or higher diarrhea	8	0.92	0.53–1.59	0.75	0
Grade 3 or higher dizziness	6	0.85	0.12–5.99	0.87	36
Grade 3 or higher fatigue	10	2.39	1.67–3.44	< 0.001	0
Grade 3 or higher headache	8	0.55	0.22–1.37	0.20	0
Grade 3 or higher nausea	9	2.57	1.57–4.22	< 0.001	0
Grade 3 or higher neutropenia	9	2.42	1.41–4.17	0.001	81
Grade 3 or higher thrombocytopenia	8	4.66	1.26–17.19	0.02	84
Grade 3 or higher vomiting	9	1.43	0.86–2.37	0.17	0

### Publication bias and sensitivity analysis of PFS

Due to the more considerable heterogeneity of PFS (I^2^ = 82.2%), we performed a publication bias test on the relationship between PFS and PARP inhibitors, which was no significant publication bias (Egger’s p = 0.241, Begg’s p = 1.00). Through the sensitivity analysis of PFS, it was found that removing each group showed excellent stability in turn (Supplementary Fig. 2 and Supplementary Fig. 3).

## Discussion

PARP inhibitors were originally developed as a radiosensitizer and chemical sensitizer for cancer treatment, but Mateo et al. pointed out in the article that some preclinical observations support the development of PARP inhibitors as a single drug for BRCA 1/2-deficient cancer [31]. Most data on BRCA 1/2 in 2005 related to the role of these genes as risk susceptibility factors for familial breast and ovarian cancer [32]. In light of this, carriers of germline BRCA 1/2 (gBRCA 1/2) mutation were the initial target population to test the PARPi (PARP inhibitors)-BRCA synthetic lethal hypothesis in the clinic [33, 34]. Since its initial approval, several other clinical trials have determined the clinical activity of PARP inhibitors in a subset of patients with somatic BRCA 1/2 mutation or another defect in the homologous recombination pathway [28, 35, 36]. With the deepening of research, it has been discovered that PARP inhibitors were not only beneficial for patients with ovarian cancer with mutation in BRCA 1/2 and HRD, but also for patients with no mutation possibly in ovarian cancer [10]. Based on this, we conducted a systematic meta-analysis to assess whether PARP inhibitors would benefit all patients with ovarian cancer (including a mutation in the BRCA 1/2 or HRD, or neither).

The results of this meta-analysis study showed that the addition of PARP inhibitors to patients with platinum-sensitive ovarian cancer had a benefit in long-term prognosis. González-Martin et al. [30] and Kaye et al. [24] have come up with conclusions consistent with our research through randomized clinical trials. Besides, the addition of PARP inhibitors had a greater benefit for ovarian cancer patients with BRCA 1/2 mutation and HRD mutation, especially for ovarian cancers with BRCA 1/2 mutation. This may be related to the fact that PARP inhibitors prevent the growth of cancer cells by inducing synthetic lethality with defects in DNA repair (such as BRCA 1/2 mutant cells) [37]. In terms of the AEs of the PARP inhibitors, the main AEs of the addition to PARP inhibitors were reflected in the blood system. Studies have shown [36] that hematological toxicity was a very common concomitant effect of PARP inhibitors, but these AEs tended to occur early after treatment begins and recover after a few months, so its overall effect was safe and tolerable.

For patients with ovarian cancer, from long-term prognosis, the addition of PARP inhibitors can benefit patients with ovarian cancer and prolong the overall disease control survival rate [14]. From the perspective of the mechanism of action of PARP inhibitors, it was found that the PARP inhibitors specifically hinder DNA repair. Therefore, we performed a subgroup analysis on whether the ovarian cancer patients had a genetic mutation and its mutation type. The analysis showed that regardless of whether the ovarian cancer patients were genetically mutated or not (BRCA 1/2 mutation or HRD mutation), the addition of PARP inhibitors could extend the prognosis of this population, and that those with BRCA 1/2 mutation or HRD mutation had significantly longer prognosis than no gene mutation. Ledermann et al. [38] and Majdak et al. [39] also reached the same conclusions as us. Studies have shown that PARP inhibitors significantly impaired the survival rate of cells with a homozygous mutation in the BRCA 1/2 gene [40, 32]. At the same time, some researchers have pointed out that cells with defective BRCA proteins cannot repair double-stranded DNA breaks through homologous recombination and rely on other approaches to repair DNA damage, especially detecting single DNA strand breaks and activating multiple effector proteins to initiate promoter repair of the PARP pathway [41], while PARP enzymes played a key role in the repair of single-strand breaks repaired by base excision [9], and inhibition of PARP in the presence of HRD leads to cells’ death because the process was called “synthetic lethality” [42], which led to an overall genetic disorder.

Most interestingly, in the absence of BRCA1/2 mutation or HRD mutation, PARP inhibitors also had clinical benefits [43, 37]. This phenomenon suggested that the role of PARP-1 in DNA damage repair may not be the sole reason for its therapeutic potential [44]. Boamah et al. [45] pointed out in the study that PARP-1 was involved in ribosome biogenesis through its enrichment in nucleoli and ADP ribosylation of several nucleolar proteins. In addition, studies have shown that PARP-1 binds to non-coding promoter-related RNA, which helps to establish silent rDNA chromatin and inhibit rRNA transcription [46]. Therefore, there were speculations that PARP inhibitors may benefit patients with ovarian cancer without a genetic mutation (BRCA1/2 mutation or HRD mutation) for two reasons [37]: (1) the role of PARP-1-RNA interactions in the nucleoli and (2) the role of PARP-1 in site-specific modification of protein substrates in ribosome biogenesis. The above reasons may explain that PARP inhibitors can also benefit patients with ovarian cancer who are not genetically mutated (BRCA1/2 mutation or HRD mutation). However, the mechanism of action of PARP inhibitors needs further study.

In terms of AEs, the use of PARP inhibitors did not significantly increase the overall risk of AEs (RR = 1.04, *p*=0.16), but in specific AEs, anemia and thrombocytopenia were the most common AEs, and their risk values were 3.40 (95% CI 1.86–6.19, *p* < 0.001) for anemia and 3.59 (95% CI 1.65–7.78, p < 0.001) for thrombocytopenia. Other AEs included decreased appetite (RR = 1.45), diarrhea (RR = 1.23), and dizziness (RR = 1.46). Among grade 3 and higher AEs, the overall risk of AEs increased (RR = 1.87, p = 0.002). Among the various AEs, the more significant increase was anemia and thrombocytopenia, and their risk values were 9.94 (95% CI 2.63–37.50, p < 0.001) and 4.46 (95% CI 1.26–17.19, p = 0.02), respectively. Other AEs were fatigue (RR = 2.39), nausea (RR = 2.57), and neutropenia (RR = 2.42). Overall, its AEs were mainly manifested in the blood system. Such AEs tended to occur early after the start of treatment and recovered after a few months. Among patients receiving niraparib, hematological of AEs accounted for the majority of grade 3 and grade 4, which were the most common cause of dose adjustment, interruption, and discontinuation [35]. Studies by Swisher et al. [36] indicated that anemia was the most common hematological toxicity among PARP inhibitors. Anemia may be a one of the targeted AEs related to PARP2 inhibition and erythropoiesis. In addition, Farrés et al. [47] pointed out that when the plasma concentration of erythropoietin increases, the loss of PARP2 can impair the differentiation of red blood cell-like progenitor cells and reduce the life span of red blood cells. We speculated that anemia, one of the toxicities of PARP inhibitors in the blood system, may be related to the above mechanism, but its mechanism had not yet been clarified and needed further study.

Regarding the AEs of thrombocytopenia, a study published by Berek et al. [48] in 2018 pointed out that patients with a baseline weight of less than 77 kg or a platelet count of less than 150,000 cells/mL had a baseline level above, and patients had more grade 3 or more thrombocytopenia events in the first month (35% vs 12%). In the case of PARP inhibitors, the cause of thrombocytopenia was associated with a reversible reduction in megakaryocyte proliferation and maturation [48]. However, thrombocytopenia usually occurred during the first month of treatment in general [36]. At the same time, FDA recommended that patients starting PARP inhibitors be tested weekly to monitor platelet concentration during the first month. In addition, neutropenia was the third most common hematological toxicity observed [36]. Although full grade neutropenia was observed in 18 to 30% of patients, niraparib (72 of 367 patients [20%]) and rucaparib (25 of 372 patients [7%]) had higher grade 3 and 4 AEs (10 of 195 patients [5%]) [28, 35, 27].

Although these grade 3 and higher AEs were relatively serious, fortunately, Oza et al. [49] reported quality of life data for patients who received niraparib as maintenance therapy; they pointed out that with the exception of nausea, all reported symptoms improved or remained stable during treatment of niraparib. At the same time, a study of quality of life on olaparib [50] eliminated the effects of AEs in the treatment and placebo groups through a unique analysis of quality-adjusted progression-free survival, which objectively significantly reduced people’s concerns about the toxic effects of PARP inhibitors. The above conclusions indicated that the AEs of PARP inhibitors were tolerable, and their duration was short [51], which would not have a serious impact on the prognosis of patients. However, these phenomena, including their mechanism of action, were not clear at present, and further research on PARP inhibitors was needed.

### Strengths and limitations

This was a meta-analysis to analyze that PARP inhibitors were associated with the prognosis of patients with ovarian cancer and AEs systematically and comprehensively. In addition, all the studies included in this meta-analysis were RCTs of phase II and phase III. However, this study showed that the sample size was insufficient and the different treatment schemes included in the study led to the diversification of the overall treatment plan of the article, which reduced the credibility of the conclusions drawn by the article. Even if we used the random effect model to increase the credibility of the conclusion as much as possible, the conclusions obtained in this article still needed to be proved by further clinical experiments.

## Conclusion

PARP inhibitors can not only prolong the prognosis of ovarian cancer patients with genetic mutation (BRCA 1/2 mutation or HRD mutation), but also prolong the prognosis of ovarian cancer patients of no gene mutation. Furthermore, PARP inhibitors were tolerable to patients when added to their current therapy, although it inevitably adds the grade 3 and higher AEs.

## Supplementary information

**Additional file 1:** Supplementary Fig. 1. Flow diagram of study inclusion and exclusion.

**Additional file 2:** Supplementary Fig. 2. Publication bias of the progression-free survival (PFS) for additional PARP inhibitors. (Egger’s) p = 0.241; (Begg’s) p = 1.0.

**Additional file 3:** Supplementary Fig. 3. Sensitivity analysis of the progression-free survival (PFS) for additional PARP inhibitors.

**Additional file 4:** Supplementary Table 1. Characteristics of included clinical trials in the meta-analysis.

## Data Availability

All the data and material are available.

## Abbreviations

PARP: Poly ADP-ribose polymerase
HRD: Homologous recombination deficiency
PFS: Progression-free survival
OS: Overall survival
AEs: Adverse events
RCTs: Randomized controlled trials
HR: Hazard ratio
RR: Risk ratio
95% CI: Confidence interval of 95%
