# Supplementary figures and images for: The efficacy and safety of the addition of poly ADP-ribose polymerase (PARP) inhibitors to therapy for ovarian cancer: a systematic review and meta-analysis
Source: World J Surg Oncol. 2020 Jul 4;18:151. doi: 10.1186/s12957-020-01931-7 (PMC7335450; doi:10.1186/s12957-020-01931-7)

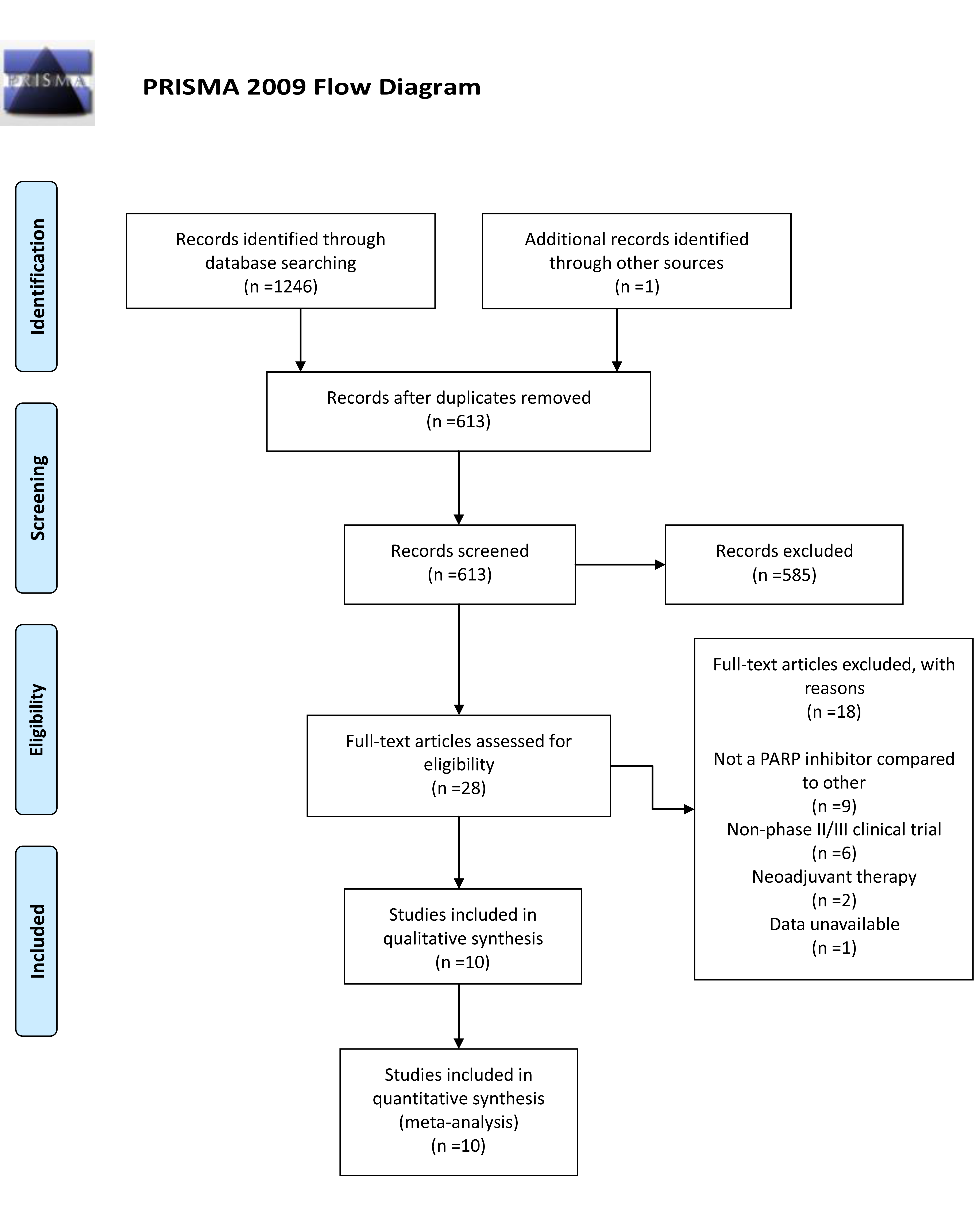

Supplement: Supplementary file 1 — Additional file 1: Supplementary Fig. 1. Flow diagram of study inclusion and exclusion. [file 12957_2020_1931_MOESM1_ESM.tif]

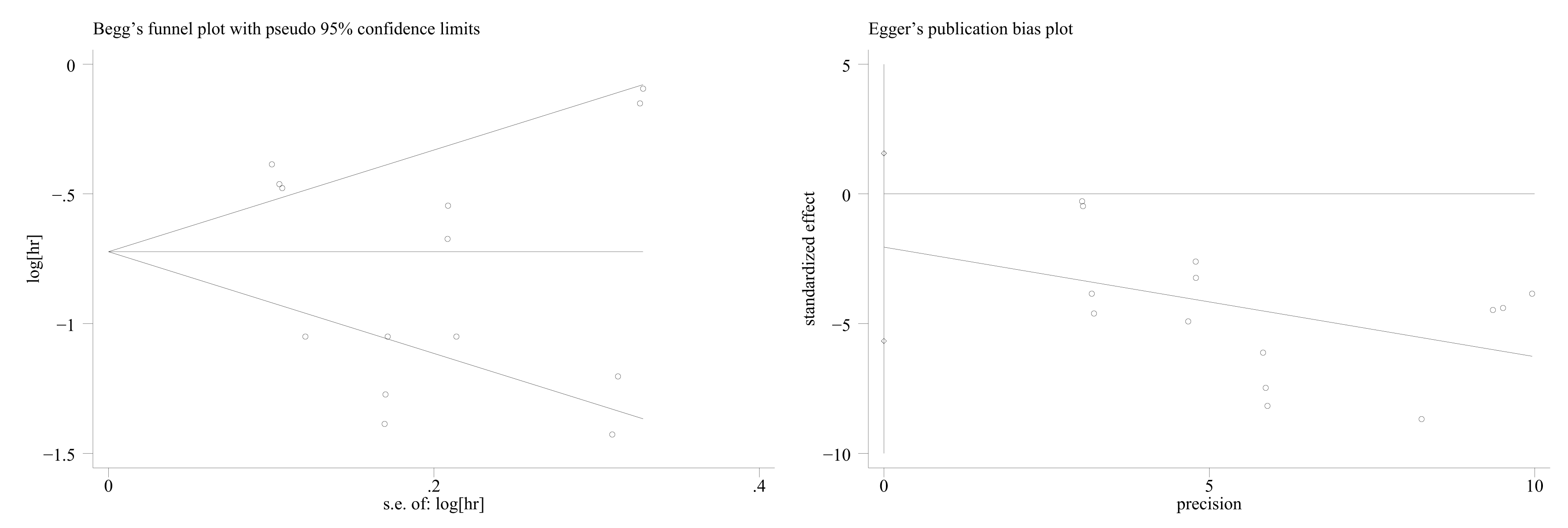

Supplement: Supplementary file 2 — Additional file 2: Supplementary Fig. 2. Publication bias of the progression-free survival (PFS) for additional PARP inhibitors. (Egger’s) p = 0.241; (Begg’s) p = 1.0. [file 12957_2020_1931_MOESM2_ESM.tif]

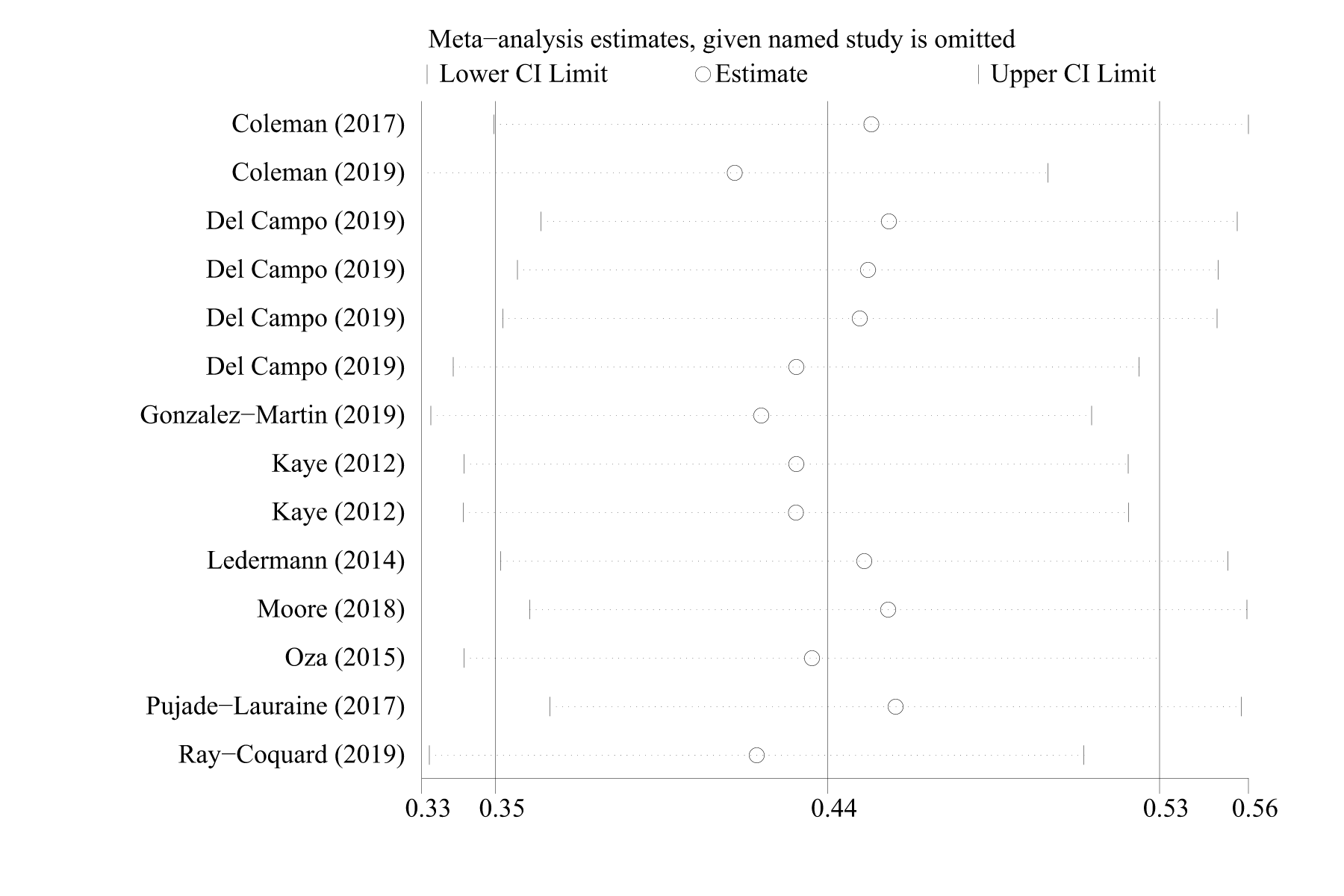

Supplement: Supplementary file 3 — Additional file 3: Supplementary Fig. 3. Sensitivity analysis of the progression-free survival (PFS) for additional PARP inhibitors. [file 12957_2020_1931_MOESM3_ESM.tif]
