# Supplementary material for: The efficacy and safety of the addition of poly ADP-ribose polymerase (PARP) inhibitors to therapy for ovarian cancer: a systematic review and meta-analysis
Source: World J Surg Oncol. 2020 Jul 4;18:151. doi: 10.1186/s12957-020-01931-7 (PMC7335450; doi:10.1186/s12957-020-01931-7)
Supplement: Supplementary file 4 — Additional file 4: Supplementary Table 1. Characteristics of included clinical trials in the meta-analysis. [file 12957_2020_1931_MOESM4_ESM.docx]

**Supplementary Table 1**. Characteristics of included clinical trials in the meta-analysis.

| Author,  year | Clinical Trials .gov, number | Phase | Therapeutic regimen | | No. of participants | Tumors mutations | Baseline | Endpoints |
| --- | --- | --- | --- | --- | --- | --- | --- | --- |
|  |  |  | PARP inhibitors | Control |  |  |  |  |
| González-Martín, 2019 | 02655016 | 3 | niraparib 300mg | placebo | 733 |  | first-line | PFS, OS |
| Ray-Coquard, 2019 | 02477644 | 3 | olaparib 300mg+bevacizumab 15mg/kg | placebo+bevacizumab15mg/kg | 806 |  | first-line | PFS |
| Kaye, 2012 | 00628251 | 2 | olaparib 200mg/400mg+pegylated liposomal doxorubicin 50mg/m^2^ | pegylated liposomal doxorubicin 50mg/m^2^ | 97 | BRCA1/2 mutation | second-line and above | PFS, OS |
| Moore, 2018 | 01844986 | 3 | olaparib 300mg | placebo | 391 | BRCA1/2 mutation | first-line | PFS, OS |
| Coleman,  2017 | 01968213 | 3 | rucaparib 600mg | placebo | 564 |  | second-line and above | PFS |
| Ledermann,  2016 | 00753545 | 2 | olaparib 400mg | placebo | 265 |  | second-line and above | PFS, OS |
| Coleman,  2019 | 02470585 | 3 | paclitaxel 175mg/m^2^/3w or 80mg/m^2^/w; AUC=6; veliparib 150mg | paclitaxel 175mg/m^2^/3w or 80mg/m^2^/w; AUC=6; placebo | 1140 |  | first-line | PFS |
| Oza, 2015 | 01081951 | 2 | paclitaxel 175mg/m^2^+carboplatin AUC=4+olaparib 200mg (combination phase); olaparib 400mg (maintenance phase) | paclitaxel 175mg/m^2^+carboplatin AUC=6 | 162 |  | second-line and above | PFS, OS |
| Pujade-Lauraine, 2017 | 01874353 | 3 | olaparib 300mg | placebo | 295 | BRCA1/2 mutation | second-line and above | PFS |
| Del Campo,  2019 | 01847274 | 3 | niraparib 300mg | placebo | 553 |  | second-line and above | PFS |

PARP, poly (ADP-ribose) polymerase; PFS, progression-free survival; OS, overall survival; AUC, area under the curve.
